# Proteomic analysis of iron acquisition, metabolic and regulatory responses of *Yersinia pestis *to iron starvation

**DOI:** 10.1186/1471-2180-10-30

**Published:** 2010-01-29

**Authors:** Rembert Pieper, Shih-Ting Huang, Prashanth P Parmar, David J Clark, Hamid Alami, Robert D Fleischmann, Robert D Perry, Scott N Peterson

**Affiliations:** 1 J. Craig Venter Institute, 9704 Medical Center Drive, Rockville, MD 20850, USA; 2 Department of Microbiology, Immunology and Molecular Genetics, University of Kentucky, Lexington, KY 40536, USA

## Abstract

**Background:**

The Gram-negative bacterium *Yersinia pestis *is the causative agent of the bubonic plague. Efficient iron acquisition systems are critical to the ability of *Y. pestis *to infect, spread and grow in mammalian hosts, because iron is sequestered and is considered part of the innate host immune defence against invading pathogens. We used a proteomic approach to determine expression changes of iron uptake systems and intracellular consequences of iron deficiency in the *Y. pestis *strain KIM6+ at two physiologically relevant temperatures (26°C and 37°C).

**Results:**

Differential protein display was performed for three *Y. pestis *subcellular fractions. Five characterized *Y. pestis *iron/siderophore acquisition systems (Ybt, Yfe, Yfu, Yiu and Hmu) and a putative iron/chelate outer membrane receptor (Y0850) were increased in abundance in iron-starved cells. The iron-sulfur (Fe-S) cluster assembly system Suf, adapted to oxidative stress and iron starvation in *E. coli*, was also more abundant, suggesting functional activity of Suf in *Y. pestis *under iron-limiting conditions. Metabolic and reactive oxygen-deactivating enzymes dependent on Fe-S clusters or other iron cofactors were decreased in abundance in iron-depleted cells. This data was consistent with lower activities of aconitase and catalase in iron-starved *vs*. iron-rich cells. In contrast, pyruvate oxidase B which metabolizes pyruvate via electron transfer to ubiquinone-8 for direct utilization in the respiratory chain was strongly increased in abundance and activity in iron-depleted cells.

**Conclusions:**

Many protein abundance differences were indicative of the important regulatory role of the ferric uptake regulator Fur. Iron deficiency seems to result in a coordinated shift from iron-utilizing to iron-independent biochemical pathways in the cytoplasm of *Y. pestis*. With growth temperature as an additional variable in proteomic comparisons of the *Y. pestis *fractions (26°C and 37°C), there was little evidence for temperature-specific adaptation processes to iron starvation.

## Background

*Yersinia pestis*, a Gram-negative bacterium, is the causative agent of the bubonic and pneumonic plague. The pathogenic lifestyle of this microbe involves two distinct life stages, one in the flea vector, the other in mammalian hosts, primarily rodents [1]. Genome sequencing and analyses have been completed for four major *Y. pestis *biovars, including the chromosome [2] and three virulence/transmission-associated plasmids [3,4] of the KIM strain, which belongs to the biovar mediaevalis. In addition to plasmid-encoded virulence factors, the genetically unstable chromosomal 102-kb *pgm *locus is also important for full virulence of *Y. pestis *in mammals and for its transmission via blocked fleas [5,6]. This locus encodes the yersiniabactin-dependent iron transport (Ybt) system and the hemin storage (Hms)-dependent biofilm system. Biofilm formation allows colonization of the flea proventriculus causing blockage which in turn induces active feeding behavior [7,8].

Efficient iron acquisition systems are critical to the ability of *Yersinia pestis *to infect, spread and grow in mammalian hosts, because iron is sequestered and is considered part of the innate host immune defence against invading pathogens [9]. The Ybt system includes a series of enzymes responsible for the siderophore's biosynthesis. Following secretion and iron chelation, the iron/yersiniabactin complex is bound by the outer membrane (OM) receptor Psn and transferred into the periplasm via TonB-dependent energy transmission. Binding of the complex to the periplasmic surface of the inner membrane-localized ATP-binding cassette (ABC) transporter YbtP/YbtQ, which contains two permease and two ATP-binding domains, initiates iron import into the cytoplasm. A functional Ybt transporter is required for bacterial infection by subcutaneous routes and important for iron acquisition in early stages of the bubonic plague in mice [10-12]. The manganese- and iron-specific ABC transporter Yfe is also important for full *Y. pestis *virulence according to data from a bubonic plague mouse model [13]. Other ABC transporters for iron (Yfu and Yiu) and hemin (Hmu) were functionally characterized, but were not found to be required for virulence in the mouse model [14-16]. The transporters Yfe and Feo serve somewhat redundant roles in ferrous iron uptake under microaerophilic growth conditions [17]. Genomic analysis suggests the existence of other transporters and OM receptors for iron/siderophores but have not been functionally characterized to date [2,18].

The ferric uptake regulator Fur is a dominant transcription factor controlling iron assimilation in many bacterial species [19]. Iron transporters, iron storage proteins and some proteins requiring iron cofactors for function feature conserved binding sites for Fur upstream of their genes. These sites are termed Fur-boxes [20]. Under iron-rich conditions, Fur binds Fe^2+^, assumes a conformation resulting in tight binding to the Fur-box and repression of gene transcription [21]. Low iron levels result in the loss of this metal ion and allosteric conformational changes in Fur that alleviate transcriptional repression. Positive regulation by Fur in Gram-negative bacteria seems to be primarily indirect via negative transcriptional control of small RNAs [22-24]. The Fur-dependent *E. coli *small RNA is termed RyhB, and two RyhB orthologs were discovered in the *Y. pestis *CO92 genome [22]. *E. coli *RyhB controls the expression of genes whose products store iron or contain iron cofactors such as heme and iron-sulfur (Fe-S) clusters [25,26]. The Fe-S cluster proteins FNR, IscR and SoxR are important global regulators [27]. Some enzymes with functions in diverse branches of cellular energy metabolism [28-30] also contain Fe-S clusters. Thus, widespread changes in the proteome and metabolome of bacteria occur due to iron starvation. In *E. coli*, the Fur regulon was reported to overlap functionally with the regulons of the catabolite repressor protein [31] and the oxidative stress regulator OxyR [32]. These overlaps suggest intriguing networks of metabolic inter-connectivity, allowing bacterial survival and growth under iron-deficient conditions. Iron homeostasis has not been thoroughly investigated to date in *Y. pestis*. Human plasma is an iron-limiting environment, and growth condition-dependent comparisons of *Y. pestis *transcriptional patterns have included growth in human plasma [33]. Many genes involved in iron acquisition and storage and the response to oxidative stress were found to be differentially expressed [33-35]. There was reasonably good agreement between the aforementioned studies and DNA microarray data comparing a Δ*fur *mutant with its Fur^+ ^parent strain [20].

Our objective was to assess iron acquisition and intracellular consequences of iron deficiency in the *Y. pestis *strain KIM6+ at two physiologically relevant temperatures (26°C and 37°C). Bacterial cultures weregrown in the absence and presence of 10 μM FeCl_3_. Cell lysis was followed by fractionation into periplasm, cytoplasm and mixed membranes. Upon pooling of two biological replicate samples for each growth condition, proteins were analysed by differential 2D gel display. Considering the high number of distinct experimental groups (fractions) and at least three required technical 2D gel replicates per experiment for meaningful statistical analyses, the rationale for sample pooling was to keep 2D gel runs at a manageable level. Sample pooling has the disadvantage that information on quantitative variability of proteins comparing biological replicates is not obtained. Proteome analysis was performed for two equivalent growth time points (13-15 h), which represented the stationary phase for iron-replete conditions (OD_600 _~2.0) and growth arrest at the OD_600 _of ~0.8 for iron-deficient conditions.

## Methods

### Bacterial strains and culture conditions

The *Y. pestis *strain KIM6+ used in this study is an avirulent derivative of the fully virulent KIM strain, which was cured of the pCD1 plasmid but retained the chromosomal *pgm *locus and the plasmids pMT1 and pPCP1 [36]. We used strain maintenance and cell growth procedures and verified the presence of the *pgm *locus on Congo Red agar as described previously [37]. Bacterial colonies were grown on tryptose blood agar at 30°C, harvested after 48 h and stored at -80°C. Aliquots of these cell stocks were used to grow 5-10 mL cultures in chemically defined PMH2 medium [14] supplemented with 10 μM FeCl_3_, followed by dilution to an OD_600 _of ~0.05 with 0.3-1 L of PMH2. PMH2 was deferrated by incubation with Chelex-100 resin overnight at 4°C [14]. Two passages of cell stocks in 10-30 mL of this medium were followed by dilution to an OD_600 _of ~0.05 with 0.3-1 L of deferrated PMH2. Overnight cell cultures (13-15 h) reached OD_600_s of *ca. *1.8-2.5 and 0.6-0.9 for iron-rich and iron-deficient cells, respectively. Chelex-100 treatment was previously shown to reduce contaminating iron levels to 0.2-0.3 μM, and replenishment of this medium with 10 μM FeCl_3 _resulted in full recovery of the normal *Y. pestis *growth rate and yield. Chelex-100 treatment likely removes some other metal ions as well. However, in contrast to iron, addition of Mn, Zn and Cu did not enhance the observed growth rate or yield. Cell pellets were harvested by centrifugation at 8,000 × *g *for 15 min at 4°C and washed with *ca. *30 volumes of 33 mM K_2_HPO_4 _(pH 7.5).

### Subcellular fractionation of *Y. pestis *cells

K_2_HPO_4_-washed *Y. pestis *cells were subjected to a lysozyme/EDTA spheroplasting method, followed by lysis of spheroplasts via sonication in a hypotonic buffer as previously described [38,39]. Soluble periplasmic and cytoplasmic fractions were exchanged into buffer A (25 mM NH_4_HCO_3_, 1 mM Na-EDTA and 1 mM benzamidine) and concentrated to 2-5 mg/mL protein at 3,000 × *g *using membrane filtration units (NMWL ~10,000). Protein concentrations were measured with the bicinchoninic acid assay, unless stated otherwise. Mixed membrane pellets were isolated from spheroplast lysates by centrifugation at 50,000 × *g *for 1 h at 4°C. These pellets were homogenized in 0.25 M sucrose, 150 mM NaCl, 10 mM Tris-OAc, pH 7.8, 5 mM Na-EDTA, 0.2 mM DTT, 10 μg/ml Leupeptin, 5 μg/ml Pepstatin, 10 μg/ml N_α_-p-Tosyl-L-arginine methyl ester and 2 mM PMSF (*ca. *10 mL/g pellet weight), and washed to remove most soluble protein contaminants. Sodium bromide (2.5 M final concentration) was added to the suspended membrane pellet, stirred for 1 h at 20°C and centrifuged at 50,000 × *g *for 1 h at 4°C. Insoluble pellets were then extracted with an ice-cold solution of 0.18 M Na_2_CO_3_, pH 11.3, 50 mM DTT, 1 mM CaCl_2_, 1 mM MgCl_2 _and 1 mM MnCl, stirred for 1 h and spun at 50,000 × *g *for 1 h at 4°C. The supernatants were not processed further. The membrane protein-enriched pellets were solubilised with 8 M urea, 2 M thiourea, 1% (w/v) amidosulfobetaine 14, 2 mM tributylphosphine and 0.5% Bio-Lyte pH 3-10 carrier ampholytes for analysis in 2D gels. Following incubation for 30 min at 20°C and centrifugation at 16,200 × *g *for 15 min, soluble aliquots of the extract, termed urea/amidosulfobetaine 14-extracted membrane (usb-MBR) fraction, were run in SDS-PAGE gels. Protein amounts were estimated from Coomassie Brilliant Blue G-250 (CBB)-stained band intensities. Integral OM proteins were more enriched than lipoproteins and integral IM proteins. The latter proteins tend to resist solubilisation or re-precipitate during the IEF separation step.

### Enzyme assays

Spectrophotometric enzyme assay were performed in 96-well microtiter plates using soluble fractions of *Y. pestis *cell lysates. Cells were harvested during the mid-exponential phase (OD_600 _~0.5-0.7) and stationary phase (OD_600 _~1.8-2.1) time points from iron-replete conditions in PMH2 medium at 26°C. Cells from two equivalent time points (OD_600 _~0.4-0.6 and OD_600 _~0.7-0.9, respectively) were harvested when growth occurred in iron-free media at 26°C. In a 100 mM NaH_2_PO_4 _buffer (pH 6.5) with 75 μg/mL lysozyme, 1 mM Na-EDTA, 1 mM PMSF and 0.1% Triton X-100, cells were subjected to pressure cycling (12 cycles of 35 kPsi for 5 sec and 0 Psi for 20 sec). After the addition of 5 mM MgCl, 10 μg/mL DNAse I and 10 μg/mL RNAse cell lysates were incubated for 45 min at 20°C and centrifuged at 16,200 × *g *for 30 min at 4°C. The supernatants were frozen at -80°C in the presence of 15% glycerol until used for enzyme assays.

Pyruvate oxidase activities were determined using sodium pyruvate and Na_3_Fe(CN)_6 _as substrates and monitoring the rate of absorbance decrease of Na_3_Fe(CN)_6 _at A_450 _(E^450 ^= 0.218 cm^-1 ^mM^-1^) while incubating at 30°C. Cell lysates were adjusted to ~0.4 mg/mL protein and assayed at pH 6.0 in 120 mM NaH_2_PO_4 _as previously reported [40], with one modification: 1% Nonidet-P40 was added to the assay buffer, because this detergent increased the activity of PoxB. Aconitase activities were determined using a coupled enzyme assay converting citrate to isocitrate and, via activity of supplemented isocitric dehydrogenase (IcdA), isocitrate to α-ketoglutarate as previously described [41] (assay kit from Cayman Chemicals, Ann Arbor, MI). The rate of absorbance increases at A_340 _(E^340 ^= 0.00622 cm^-1 ^μM^-1^) due to formation of the IcdA product NADPH was monitored while incubating at 37°C. To increase the pH and stabilize aconitase, crude extracts were exchanged into 50 mM Tris-HCl (pH 7.5), 0.6 mM MnCl_2 _and 2 mM sodium citrate, and adjusted to ~0.5 mg/mL protein. To distinguish the aconitase/IcdA activity from other NADP^+^-dependent oxidoreductive enzymes, the aconitase inhibitor oxalomalate was added at a 18.7 mM concentration to the assay mixture. Catalase activities were determined assessing the peroxidatic function with methanol as a substrate and formaldehyde as a product in the presence of optimal concentrations of H_2_O_2 _at 22°C. Crude extracts were diluted to ~0.15 mg/mL protein, and formaldehyde was measured colorimetrically at A_540 _in an endpoint assay via addition of the chromogen 4-amino-3-hydrozino-5-mercapto-1,2,4-triazole, as previously described [42] (assay kit from Cayman Chemicals). Superoxide dismutase (SOD) activities were determined using a coupled enzyme assay measuring the dismutation of the superoxide radical formed by xanthine oxidase at 22°C. The reaction was coupled to the conversion of a tetrazolium salt to formazan whose absorbance was measured at A_450 _in an endpoint assay as described [43] (assay kit from Cayman Chemicals). Cell lysates were diluted to ~1.1 μg/mL protein to measure SOD activities.

### Protein separation and differential display in 2D gels

Equal protein amounts from two biological replicates of periplasmic and cytoplasmic fractions were combined and diluted in a 1:5 to 1:10 ratio with RB buffer, which contained 8 M urea, 2 M thiourea, 4% (w/v) CHAPS, 18 mM DTT and 0.5% (v/v) Bio-Lyte pH 3-10 carrier ampholytes. Equal protein amounts from solubilised biological replicates of mixed membrane fractions were also combined. The rationale for sample pooling is described at the end of the 'Background' section. *Circa *75 μg protein for Sypro Ruby^®^-stained gels and 130 μg for Coomassie Brilliant Blue G250 (CBB)-stained gels were loaded via rehydration loading onto 24 cm IPG gel strips (pH ranges 4-7 and 3-10) and separated in the 1^st ^dimension as previously described [39]. Established methods were also used for 2^nd ^dimension slab gel electrophoresis (25 × 19.5 × 0.15 cm), gel staining with CBB, scanning and gel image import into the analysis software Proteomweaver v.4.0 [44]. The scope of differential 2D display analysis was extensive, with three subcellular fractions and four growth conditions (fourteen experimental groups for seven group-to-group comparisons, among them two analyses for the periplasmic fraction with 2D gels in the pH ranges 4-7 and 6.5-10). Software-assisted gel image analysis included spot matching, pre-match and post-match spot normalization and spot intensity averaging. The analysis mode did not require internal standards for spot normalization. The Mann-Whitney Test was used for statistical significance analysis of spot abundance changes. It is a non-parametric two sample distribution-free t-test and assesses whether two independent samples of observations come from the same distribution: , where n1 and n2 are numbers of observations in the samples and R1 is the sum of the ranks of the observations in sample 1. P-values determined by this test are based on 3 ≤ n ≤ 5 observations, which reflect 2D spot intensity data from an equal number of replicate gels. Provided that spot abundance ratios were ≥1.5, p-values < 0.02 were considered statistically significant. Protein spots observed in only one the two sample groups required spot matches among at least three technical replicates, but p-values could not be determined.

### Mass spectrometry and bioinformatic protein analysis

Nearly all spots derived from 2D gels of the three *Y. pestis *subcellular fractions were analyzed by mass spectrometry (MS) two or more times. This was necessary in order to link each spot abundance change unambiguously to identification of a distinct protein; limitation of spot resolution in 2D gels is a known problem when the analyzed samples are highly complex. Prerequisites for confident spot identification were known protein identities of surrounding spots with equal or higher abundance and the comparison of Mascot scores in those spots. Methods for spot cutting and protein digestion with trypsin were reported previously [45]. Peptide digests were analyzed using a MALDI-TOFTOF mass spectrometer (4700 Proteomics Analyzer, Applied Biosystems) and a nano-electrospray LC-MS/MS system (LTQ ion trap mass spectrometer, Thermo-Finnigan, San Jose, CA) equipped with an Agilent 1100 series solvent delivery system (Agilent, Palo Alto, CA). Reversed phase peptide separations for LC-MS/MS analysis were performed at nanoflow rates (350 nL/min). Technical details of MS and MS^2 ^analysis methods have been described [45]. The data were searched against the latest release of the *Y. pestis *KIM strain subset of the NCBInr database, using the Mascot searching engine v.2.1 (Matrix Science, London, UK). Carbamidomethyl was invariably selected as a fixed modification and one missed tryptic cleavage was allowed. MALDI search parameters (+1 ions) included mass error tolerances of ± 100 ppm for peptide precursor ions and ± 0.2 Da for fragment ions. LTQ ion trap search parameters (+1, +2 and +3 ions) included mass error tolerances of ± 1.4 Da for peptide precursor ions and ± 0.5 Da for fragment ions. Protein identifications were accepted as significant when Mascot protein scores >75 were obtained. Using a randomized decoy database, setting a default significance threshold of 0.05 in the Mascot algorithm and requiring two peptide e-values < 0.1 per protein identification, the false positive rate of proteins by LC-MS/MS was estimated to be <0.5%. Bioinformatic predictions of *Y. pestis *KIM iron transporters and binding proteins, of transmembrane domains, of protein export signal motifs and of β-barrel OM protein motifs were derived from the algorithms utilized in TransportDB http://www.membranetransport.org, TMHMM and SignalP http://www.cbs.dtu.dk/services and PRED-TMBB [46], respectively.

## Results

### Using subcellular fractionation and differential 2D gel display to assess the response of *Y. pestis *to iron starvation

Three subcellular fractions of the *Y. pestis *strain KIM6+, a periplasmic, a cytoplasmic and a membrane fraction enriched in integral OM proteins, were isolated from cells cultured at two growth temperatures (26°C and 37°C), without FeCl_3 _or supplemented with 10 μM FeCl_3_. Data published on the periplasmic and membrane proteomes of this strain facilitated the study [39,44,47]. Extensive protein identification efforts from soluble cytoplasmic fractions were performed for this study. We estimated the coverage of subcellular proteomes by comparing predicted localizations of experimentally identified proteins with those *in silico *assigned to the ORFs annotated in the *Y. pestis *KIM genome. The algorithm used here was PSORTb. Limiting this to the proteins clearly assigned to distinct 2D gel spots, the coverage was roughly 25% for the periplasm, 20% for the cytoplasm, 1% for the IM and 25% for the OM. The prediction of subcellular proteomes is incomplete because assignments are not made for all ORFs (*e.g.*, 45% of the 4086 *Y. pestis *KIM ORFs using PSORTb). Many proteins were not profiled quantitatively. However, subcellular fractionation allowed us to increase the number of surveyed proteins and the dynamic range of abundance measurements.

Proteome profiles derived from iron-starved and iron-replete growth conditions, often abbreviated as '-Fe and +Fe conditions' from here on, were compared. When cells were harvested, they were in the stationary phase for at least 3 h (+Fe conditions) or near complete growth arrest due to the lack of iron (-Fe conditions). This is visualized in growth curves at the temperatures of 37°C and 26°C provided in the graphics of Additional File 1. Cells grown in the absence of iron at 37°C consistently reached a 10-20% higher OD_600 _than those grown at 26°C. Earlier growth time points (exponential phase) would have been of interest, but were not included due to already extensive proteomic profiling efforts. Our rationale was that the greater difference in cell doubling times during the exponential phase (-Fe *vs. *+Fe) would have confounded identification of iron starvation-specific protein changes more than that for the late growth stage.

Differential display experiments were focused on the pH range 4-7 in 2D gels because the majority of mature proteins have pI values ranging from 4 to 7. The removal of basic N-terminal signal sequences from exported proteins, which are displayed in the periplasmic and mixed membrane fractions, often result in a shift towards more acidic pIs. Few integral IM proteins, typically those with low M_r _values, were quantitatively profiled because TMD proteins are too hydrophobic to be sufficiently solubilised or resolved as spots in 2D gels. Periplasmic fractions consistently showed contamination with cytoplasmic proteins which was attributed to partial lysis of *Y. pestis *spheroplasts during the fractionation. The outcome of this cross-contamination was a moderately decreased depth of analysis for periplasmic proteins. Of nearly 250 statistically significant spot abundance changes with confident protein identifications, observed at 26°C and/or 37°C, some were associated with spot trains. Particularly the 2D profile of the usb-MBR fraction featured extensive spot trains. Based on MS^2 ^data from MALDI-TOFTOF and ESI-ion trap experiments, we have evidence that Asn and Gln amino acid side chain deamidations, which lead to acidic 2D spot shifts of proteins, account for many of the spot trains [48]. The Table of Additional File 2 lists all significant spot abundance changes (-Fe *vs. *+Fe conditions). Comprehensive MS and MS^2 ^datasets are provided in the Table of Additional File 3. The concise protein lists in the Tables 1, 2 and 3 are of particular interest in the context of iron homeostasis. Only if protein abundance ratios differed substantially comparing the -Fe *vs. *+Fe datasets at 26°C and 37°C, the temperature dependency was pointed out in the following paragraphs.

**Table 1:** Abundance differences of *Y. pestis *proteins profiled in periplasmic fractions of iron-rich vs. iron-starved cells

Spot No a)	Gene locus b)	gene name c)	Protein description c)		Subc. Loc. d)	Fur/RyhB e)	Mascot Score f)	exp Mr (Da)	exp pI	26°C, Vs (-Fe) g)	26°C, Vs (+Fe) h)	26-ratio -Fe/+Fe i)	26°C P-value j)	37-ratio -Fe/+Fe k)
53	y0028	malE	periplasmic maltose-binding protein		PP		2150	43937	5.53	0.72	5.98	0.121	0.000	0.760
54	y0137	degQ	serine endoprotease		PP		1077	55588	6.43	0.39	0.11	2.41	0.0177	0.900
55	y0291	-	putative tospovirus resistance protein D		U		486	18721	5.44	2.05	0.47	4.320	0.000	N.D.
56	y0541	hmuT	hemin-binding periplasmic protein		PP	Fur	228	27164	5.85	0.46	0.11	4.328	0.000	> 20
57	y0542	hmuS	hemin uptake system component		U	Fur	989	38188	5.56	0.53	0.19	2.780	0.000	2.091
58	y0869	cybC	cytochrome b(562)		PP	Fur	626	5035	5.64	0.13	0.03	4.746	N.D.	3.160
59	y0964	frsA	fermentation/respiration switch protein		U		586	51326	5.98	0.15	0.07	2.208	0.000	1.875
60	y1128	bglX	putative beta-glucosidase		PP		2324	81506	5.43	3.01	0.52	5.822	0.000	1.740
61	y1189	gltI	solute-binding periplasmic protein of glutamate/aspartate ABC transporter		PP		2512	35927	7.20	0.41	2.91	0.141	0.005	N.D.
62	y1223	nrdE	ribonucleoside-diphosphate reductase 2, alpha subunit		U	Fur	198	79914	6.32	0.03	-	> 20	N.D.	N.D.
63	y1222	nrdF	ribonucleoside-diphosphate reductase 2, beta chain		U	Fur	561	39335	5.11	0.77	-	> 20	N.D.	> 20
64	y1430	-	putative putative periplasmic iron-binding signal peptide protein		U		3359	41211	6.09	-	0.57	< 0.05	N.D.	< 0.05
65	y1526	yfuA	putative solute-binding protein for iron ABC transporter		PP	Fur	1979	39620	6.65	2.36	1.46	1.618	0.061	N.D.
66	y1607	hisJ	histidine-binding periplasmic protein of high-affinity histidine transport system		PP		1494	31529	5.01	0.29	0.93	0.309	0.000	0.350
67	y1744	-	hypothetical protein y1744		CY		324	5183	5.92	0.38	-	> 20	N.D.	4.510
68	y1897	yfeA	periplasmic-binding protein for iron and manganese ABC transporter		CM	Fur	1201	31395	5.80	2.87	0.63	4.576	0.000	4.780
69	y1936	sufC	iron-sulfur cluster assembly protein SufC, ATPase component		ML	Fur	726	28460	5.10	0.16	0.02	7.514	0.000	> 20
70	y1937	sufD	cysteine desulfurase activator complex subunit SufD		U	Fur	369	60476	6.76	0.06	-	> 20	N.D.	N.D.
71	y2358	hmsF	hemin storage system, HmsF protein		U		542	61790	5.47	0.40	0.12	3.467	0.000	1.480
72	y2368	-	putative ferrous iron transport protein		U		532	13556	5.29	1.71	1.64	1.030	0.390	2.330
73	y2394	ybtS	anthranilate synthase		CY	Fur	1323	50265	5.82	1.65	0.36	4.538	0.000	> 20
74	y2401	ybtU	thiazolinyl-S-HMWP1 reductase of Ybt system		U	Fur	351	48765	6.63	0.33	0.11	3.057	0.006	N.D.
76	y2403	ybtE	salicyl-AMP ligase		CY	Fur	1205	58276	5.43	2.04	0.31	6.660	0.000	7.060
77	y2451	efeO	putative ferrous iron transport protein		U		998	38614	4.96	1.71	0.90	1.896	0.000	1.274
78	y2638	ysuG	siderophore biosynthetic protein of the Ysu system		U	Fur	182	77918	5.36	0.06	-	> 20	N.D.	N.D.
79	y2662	mglB	periplasmic D-galactose-binding ABC transport protein		PP		1440	33113	5.40	0.51	1.53	0.330	0.000	0.251
80	y2828	pheA	putative chorismate mutase		PP		630	14433	5.88	0.86	0.05	19.293	0.000	2.817
81	y2842	-	putative periplasmic binding protein of iron/siderophore ABC transporter		U		1096	51189	5.97	0.62	1.87	0.332	0.000	0.501
82	y2875	yiuA	solute-binding periplasmic protein of iron/siderophore ABC transporter		U	Fur	1690	46030	6.69	0.73	0.37	1.957	0.002	N.D.
83	y3037	modA	molybdate-binding periplasmic protein of molybdate ABC transporter		PP		2136	27031	5.55	0.17	0.72	0.234	0.000	2.089
84	y0815	sodC	periplasmic superoxide dismutase (Cu-Zn)		PP		695	16562	7.54	0.55	0.63	0.89	0.4490	N.D.
85	y3165	ptr	protease III		PP		1794	96878	5.60	2.71	1.86	1.454	0.001	1.032
86	y3676	-	putative type VI secretion system protein		CY		375	50035	4.81	0.29	-	> 20	N.D.	N.D.
87	y3772	lsrB	putative periplasmic autoinducer II-binding protein		U		917	36377	6.30	0.31	1.96	0.159	0.000	N.D.
88	y3812	dsbA	protein disulfide isomerase I		PP		1587	22454	5.91	2.57	1.18	2.176	0.000	0.910
89	y3825	dppA	periplasmic dipeptide transport protein of ABC transporter		PP		1253	54903	5.52	0.68	2.46	0.277	0.000	0.696
90	y3837	yhjJ	predicted zinc-dependent peptidase		U		1215	62177	5.10	0.44	0.17	2.613	0.000	0.720
91	y3956	crp	cAMP-regulatory protein		CY		220	26494	7.82	0.06	-	> 20	N.D.	N.D.
92	y3977	fkpA	FKBP-type peptidyl-prolyl cis-trans isomerase		PP		2031	33670	6.94	5.50	3.45	1.594	0.007	N.D.
93	y4125	-	putative solute-binding periplasmic protein precursor for ABC transporter		PP		2766	30250	6.27	6.09	3.67	1.661	0.001	2.264

**Figure 1:**
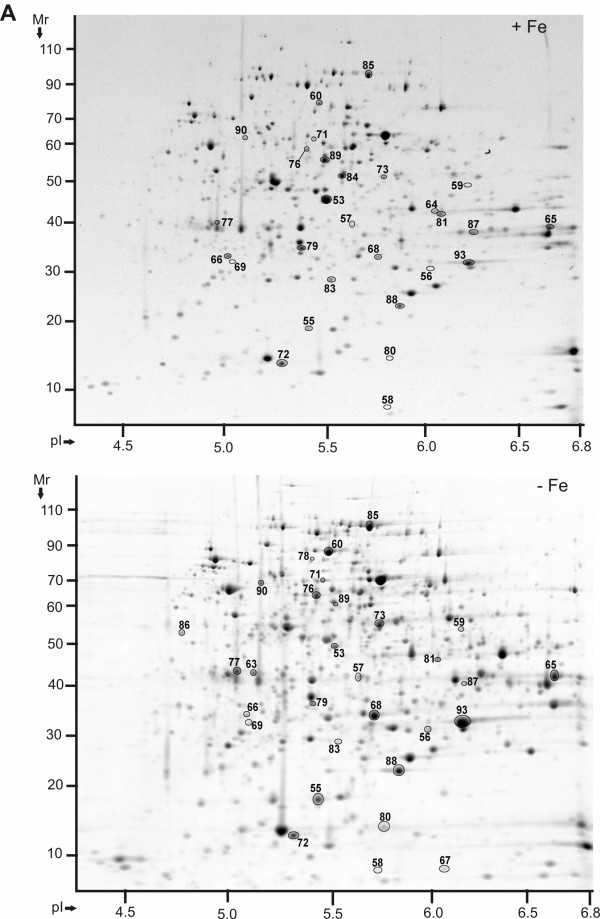
**Protein display in 2D gels of *Y. pestis *KIM6+ periplasmic fractions in the pI range 4-7 (-Fe *vs. *+Fe conditions)**. Proteins were derived from cell growth in the presence of 10 μM FeCl_3 _at 26°C (top) or the absence of FeCl_3 _at 26°C (bottom). Gels (20 × 25 cm) were stained with Coomassie Brilliant Blue G250 (CBB), with five gel replicates representing each group, and subjected to differential display analysis using the software Proteomweaver v.4.0. Protein assignment to a spot required validation by MS data from at least two representative gels. The denoted spot numbers are equivalent to those listed in Table 1 with their '-Fe *vs. *+Fe' protein abundance ratios and other data.

**Figure 2:**
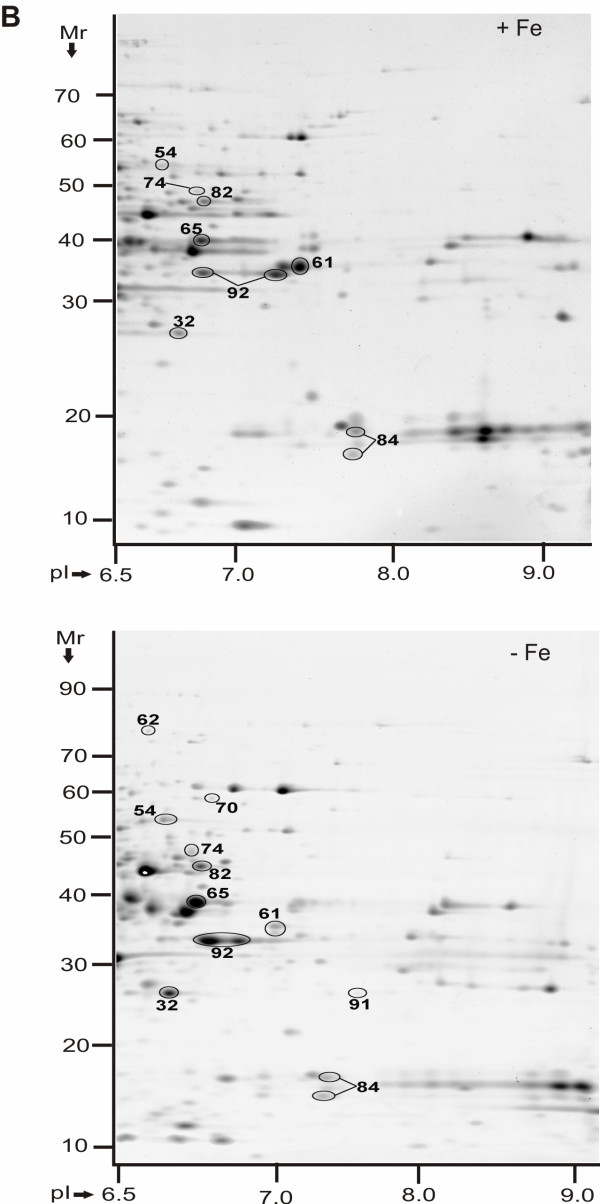
**Protein display in 2D gels of *Y. pestis *KIM6+ periplasmic fractions in the pI range 6.5-9 (-Fe *vs. *+Fe conditions)**. Proteins were derived from cell growth in the presence of 10 μM FeCl_3 _at 26°C (top) or absence of FeCl_3 _at 26°C (bottom). Gels (20 × 25 cm) were stained with CBB, with three gel replicates representing each group, and subjected to differential display analysis using the software Proteomweaver v.4.0. Protein assignment to a spot required validation by MS data from at least two representative gels. The denoted spot numbers are equivalent to those listed in Table 1 with their '-Fe *vs. *+Fe' protein abundance ratios and other data.

a) spot number as denoted in Figures 1 and 2; b) protein accession number and locus tag as listed in *Y. pestis *KIM genome database (NCBI); c) gene name and protein description from the KIM database or a conserved *E. coli *K12 ortholog http://www.ecocyc.org, if >65 pct. sequence identity; d) subcellular localization based on PSORTb data: CY, cytoplasm; ML: multiple localizations; CM: inner membrane; PP, periplasm; U: unknown; e) proven or putative regulation by Fur or a Fur-dependent small RNA (e.g. RyhB); f) highest Mascot score for a protein from LC-MS/MS or MALDI data; g) Vs (-Fe): average spot volume (n ≥ 3) in 2D gels for iron-depleted growth conditions at 26°C as shown in Figures 1-2; h) Vs (+Fe): average spot volume (n ≥ 3) in 2D gels for iron-supplemented growth conditions at 26°C; i) spot volume ratio (-Fe/+Fe) at 26°C, N.D.: not determined; -: no spot detected; j) two-tailed t-test p-value for spot abundance change at 26°C; 0.000 stands for < 0.001; k) average spot volume ratio (-Fe/+Fe) at 37°C; additional data for the statistical spot analysis at 37°C are part of Additional Table 1. exp.pI/Mr = experimental pI and Mr values.

**Table 2:** Abundance differences of *Y. pestis *proteins profiled in membrane fractions of iron-rich vs. iron-starved cells

Spot No a)	Gene locus b)	gene name c)	Protein description c)		Subc. Loc. d)	Fur/RyhB e)	Mascot Score f)	exp Mr (Da)	exp pI	26°C, Vs (-Fe) g)	26°C, Vs(+Fe) h)	26-ratio -Fe/+Fe i)	26°C P-value j)	37-ratio -Fe/+Fe k)
94	y0032	lamB	Maltoporin		OM		331	48645	[4.95 - 5.09]	0.76	1.49	0.516	0.000	1.27
95	y0543	hmuR	hemin outer membrane receptor		OM	Fur	1064	76570	5.05	0.25	0.10	2.600	0.000	4.665
96	y0850	-	putative iron/chelate outer membrane receptor		OM	Fur	57	70302	[5.5 - 6.0]	1.54	0.22	6.978	0.000	2.430
97	y1355	-	hypothetical inner membrane protein y1355		U		53	22715	5.59	0.32	0.57	0.560	0.000	0.820
98	y1577	fadL	long-chain fatty acid transport protein (OM receptor)		OM		1008	51392	[4.77 - 4.87]	0.37	0.81	0.460	0.000	0.370
99	y1632	nuoC	NADH dehydrogenase I chain C, D		CY		654	68079	[5.79 - 5.9]	0.07	0.18	0.367	0.000	0.578
100	y1682	ompX	outer membrane protein X		OM		389	18271	5.31	5.65	3.08	1.859	0.000	0.557
101	y1919	arnA	bifunctional UDP-glucuronic acid decarboxylase/UDP-4-amino-4-deoxy-L-arabinose formyltransferase		U		346	72392	[5.86 - 5.92]	0.76	0.20	3.748	0.000	> 20
102	y2404	psn	pesticin/yersiniabactin outer membrane receptor		OM	Fur	148	67582	[5.20 - 5.45]	6.80	1.46	4.862	0.000	2.656
103	y2556	fcuA	ferrichrome receptor, TonB dependent		OM	Fur	801	76097	[5.64 - 5.94]	0.20	0.18	1.070	0.710	0.860
104	y2633	ysuR	outer membrane iron/siderophore receptor		OM	Fur	202	73135	6.30	0.11	0.04	2.790	0.001	N.D.
105	y2735	ompA	outer membrane porin A, N-t. fragment		OM		686	34018	[5.52 - 5.75]	5.05	0.70	7.245	0.000	3.390
106	y2872	yiuR	putative iron/siderophore outer membrane receptor		OM	Fur	133	67256	5.55	0.65	0.29	2.260	0.000	N.D.
107	y2966	ompC	outer membrane porin protein C		OM		1110	43707	[4.78 - 4.88]	2.18	1.45	1.500	0.010	0.487
108	y2980	yfaZ	hypothetical protein y2980		CM		96	20054	5.48	0.30	0.66	0.459	0.000	0.202
109	y2983	phoE	putative outer membrane porin		OM		65	41703	[4.94 - 5.22]	-	14.60	< 0.05	N.D.	< 0.05
110	y3674	-	putative type VI secretion system protein		U		350	63614	[5.52 - 5.58]	0.72	0.44	1.620	0.002	N.D.

**Figure 3:**
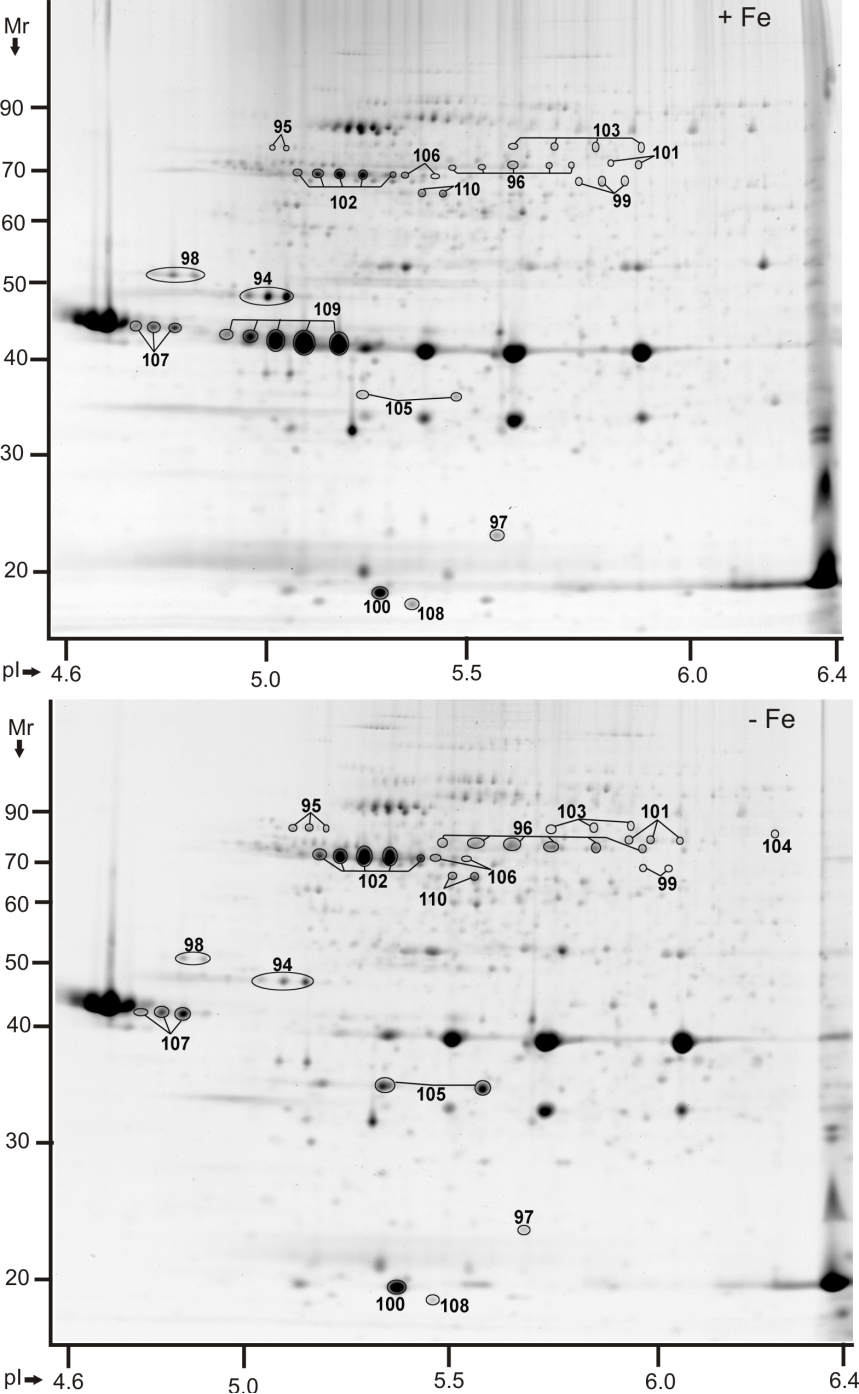
**Protein display in 2D gels of *Y. pestis *KIM6+ membrane fractions in the pI range 4-7 (-Fe *vs. *+Fe conditions)**. Proteins were derived from cell growth in the presence of 10 μM FeCl_3 _at 26°C (top) or absence of FeCl_3 _at 26°C (bottom). Gels (20 × 25 cm) were stained with CBB, with five gel replicates representing each of the groups, and subjected to differential display analysis using the software Proteomweaver v.4.0. Protein assignments to a spot (or a spot train) required validation by MS data from at least two representative gels. The denoted spots and spot trains are equivalent to those listed in Table 2 with their '-Fe *vs. *+Fe' protein abundance ratios and other data.

a) spot number as denoted in Figure 3; b) protein accession number and locus tag as listed in *Y. pestis *KIM genome database (NCBI); c) gene name and protein description from the KIM database or a conserved *E. coli *K12 ortholog http://www.ecocyc.org, if >65 pct. sequence identity; d) subcellular localization based on PSORTb data: CY, cytoplasm; CM: inner membrane; OM: outer membrane; U: unknown; e) proven or putative regulation by Fur or a Fur-dependent small RNA (e.g. RyhB); f) highest Mascot score for a protein from LC-MS/MS or MALDI data; g) Vs (-Fe): average spot volume (n ≥ 3) in 2D gels for iron-depleted growth conditions at 26°C as shown in Figure 3; h) Vs (+Fe): average spot volume (n ≥ 3) in 2D gels for iron-supplemented growth conditions at 26°C; i) spot volume ratio (-Fe/+Fe) at 26°C, N.D.: not determined; -: no spot detected; j) two-tailed t-test p-value for spot abundance change at 26°C, 0.000 stands for < 0.001; k) average spot volume ratio (-Fe/+Fe) at 37°C; additional data for the statistical spot analysis at 37°C are part of Additional Table 1. Experimental pI values span a pH range because proteins were visualized as spot trains.

**Table 3:** Abundance differences of *Y. pestis *proteins profiled in cytoplasmic fractions of iron-rich vs. iron-starved cells

Spot No a)	Gene locus b)	gene name c)	Protein description c)		Subc. Loc. d)	Fur/RyhB e)	Mascot Score f)	exp Mr (Da)	exp pI	26°C, Vs (-Fe) g)	26°C, Vs (+Fe) h)	26-ratio -Fe/+Fe i)	26°C P-value j)	37-ratio -Fe/+Fe k)
1	y0015	aceB	malate synthase A		CY	Fur	688	63974	5.86	0.06	1.73	0.036	0.000	0.421
2	y0016	aceA	isocitrate lyase		CY		741	54571	5.47	0.38	4.19	0.090	0.000	0.408
3	y0047	glpK	glycerol kinase		CY		828	60235	6.01	0.07	0.33	0.198	0.000	0.570
4	y0320	oxyR	DNA-binding transcriptional regulator OxyR		CY		510	36649	5.82	0.49	0.40	1.237	0.004	0.791
5	y0548	metF2	putative methylenetetrahydrofolate reductase		U		321	31848	5.73	1.77	1.06	1.677	0.000	0.543
6	y0617	frdA	fumarate reductase, anaerobic, flavoprotein subunit		PP		437	80764	5.77	-	0.23	< 0.05	N.D.	0.339
7	y0668	mdh	malate dehydrogenase		ML		2170	34545	5.55	1.03	1.80	0.576	0.001	1.253
8	y0771	acnB	aconitate hydrase B		CY	RyhB	1408	95757	5.13	0.22	0.98	0.220	0.000	0.229
9	y0801	erpA	iron-sulfur cluster insertion protein ErpA		U		76	10730	4.41	1.41	0.57	2.492	0.008	1.260
10	y0818	cysJ	sulfite reductase subunit alpha		U		340	72332	4.97	-	0.20	< 0.05	N.D.	< 0.05
11	y0854	fumA	fumarase A		CY	RyhB	255	68184	6.02	-	0.50	< 0.05	N.D.	< 0.05
12	y0870	katY	catalase; hydroperoxidase HPI(I)		U		768	78569	6.32	0.11	0.48	0.231	0.000	0.081
13	y0888	luxS	predicted S-ribosylhomocysteinase		CY		670	19733	5.46	0.79	0.30	2.617	0.000	2.164
14	y0988	ahpC	putative peroxidase		CY		898	24298	5.75	5.02	6.10	0.823	0.202	1.376
15	y1069	ymt	murine toxin		U		7052	67771	5.64	13.61	9.61	1.415	0.143	1.359
16	y1069	ymt	murine toxin, C-t. fragment		U		245	33893	5.30	0.89	0.19	4.634	0.000	N.D.
17	y1069	ymt	murine toxin, N-t. fragment		U		164	39074	6.11	0.84	0.17	4.860	0.000	N.D.
18	y1208	fur	ferric uptake regulator		CY		95	13425	6.16	0.11	0.17	0.651	0.055	N.D.
19	y1282	yfiD	formate acetyltransferase, glycyl radical cofactor GrcA		CY		521	13866	4.75	1.27	2.27	0.560	0.000	0.456
20	y1334	iscS	selenocysteine lyase/cysteine desulfurase		U		408	51519	5.96	-	-	N.D.	N.D.	0.59
21	y1339	hscA	chaperone protein HscA		CY		384	49149	5.53	-	-	N.D.	N.D.	0.48
22	y1342	pepB	aminopeptidase B		U		855	52355	5.69	0.12	0.75	0.153	0.000	0.681
23	y1452	ypeA	predicted acyltransferase		CY		188	12771	4.83	0.39	0.14	2.844	0.000	3.300
24	y1677	dps	DNA starvation/stationary phase protection protein		U		724	14844	5.94	0.27	0.80	0.337	0.000	0.808
25	y1791	pepT	putative peptidase T		CY		310	51106	5.89	-	0.18	< 0.05	N.D.	N.D.
26	y1802	icdA	isocitrate dehydrogenase, specific for NADP+		CY		459	53760	5.46	0.92	1.80	0.511	0.002	1.238
27	y1934	sufA	iron-sulfur cluster assembly scaffold protein SufA		U	Fur	156	13330	4.48	0.13	-	> 20	N.D.	2.170
28	y1935	sufB	cysteine desulfurase activator complex subunit SufB		U	Fur	330	70431	4.69	0.25	0.06	4.022	0.000	3.836
29	y1938	sufS	selenocysteine lyase		U	Fur	369	46479	5.55	0.65	0.15	4.294	0.000	2.420
30	y1944	pykF	pyruvate kinase I		CY		525	62400	5.93	0.38	1.23	0.309	0.525	1.265
31	y1951	sodB	superoxide dismutase, iron		U	RyhB	285	21541	5.75	0.16	0.94	0.172	0.000	>20
32	y1968	gst	glutathionine S-transferase		CY		1326	25438	6.25	3.15	2.14	1.471	0.054	1.247
33	y1990	tpx	thiol peroxidase		U		479	18655	5.13	3.02	3.06	0.986	0.816	1.198
34	y2063	acnA	aconitate hydratase A		CY	RyhB	565	97825	6.08	-	0.22	< 0.05	N.D.	< 0.05
35	y2255	yebC	hypothetical protein y2255		U		219	39957	4.74	0.11	0.40	0.285	0.000	0.777
36	y2524	ftnA	ferritin iron storage complex protein		CY	RyhB	223	14143	4.99	2.67	1.61	1.656	0.000	1.275
37	y2790	pflB	formate acetyltransferase 1		CY		804	80979	5.49	0.63	1.38	0.454	0.000	0.980
38	y2802	trxB	thioredoxin reductase		ML		702	37892	5.21	0.96	0.99	0.967	0.446	1.037
39	y2821	poxB	pyruvate oxidase		CY		448	67362	5.91	1.89	0.33	5.722	0.000	3.710
40	y2981	katE	catalase; hydroperoxidase HPII(III)		CY	RyhB	481	66313	6.09	0.04	1.20	0.032	0.000	0.113
41	y3064	sucD	succinyl-CoA synthetase, alpha subunit		CY		597	33015	6.04	0.33	0.91	0.363	0.000	0.472
42	y3067	sucA	2-oxoglutarate dehydrogenase (decarboxylase component)		CY		1153	102739	5.98	-	0.43	< 0.05	N.D.	0.277
43	y3069	sdhA	succinate dehydrogenase, flavoprotein subunit		ML	RyhB	965	75497	5.56	0.05	0.21	0.248	0.000	0.207
44	y3142	fldA3	predicted flavodoxin		CY		267	11842	4.37	0.93	0.39	2.395	0.003	1.502
45	y3499	yqhD	NADP-dependent dehydrogenase		CY		369	46727	5.76	0.35	1.922	0.179	0.001	1.404
46	y3600	uxaC	D-glucuronate/D-galacturonate isomerase		U		842	56072	5.75	0.09	-	> 20	N.D.	2.383
47	y3673	hcp1	hemolysin-coregulated protein		U		508	14459	5.16	8.35	4.38	1.908	0.001	N.D.
48	y3675	-	putative type VI secretion protein		CY		392	25923	4.62	0.43	0.16	2.735	0.001	N.D.
49	y3802	bipA	putative GTP-binding factor		CY		435	82945	5.27	-	-	N.D.	N.D.	4.096
50	y3966	tauD	taurine dioxygenase		U		228	40946	6.12	0.50	0.16	3.129	0.001	N.D.
51	y3988	bfr	bacterioferritin, iron storage and detoxification protein		CY	RyhB	143	17087	4.92	0.22	0.29	0.779	0.006	0.927
52	y4080	sodA	superoxide dismutase, manganese		U		597	25405	5.86	4.11	5.10	0.805	0.074	0.877
75	y2402	ybtT	yersiniabactin thioesterase		U	Fur	123	34389	5.88	0.10	-	> 20	N.D.	12.08
111	y3066	sucB	2-oxoglutarate dehydrogenase (dihydrolipoyltranssuccinase E2 component)		CY		789	58844	5.38	0.29	0.76	0.38	0.0468	0.38

**Figure 4:**
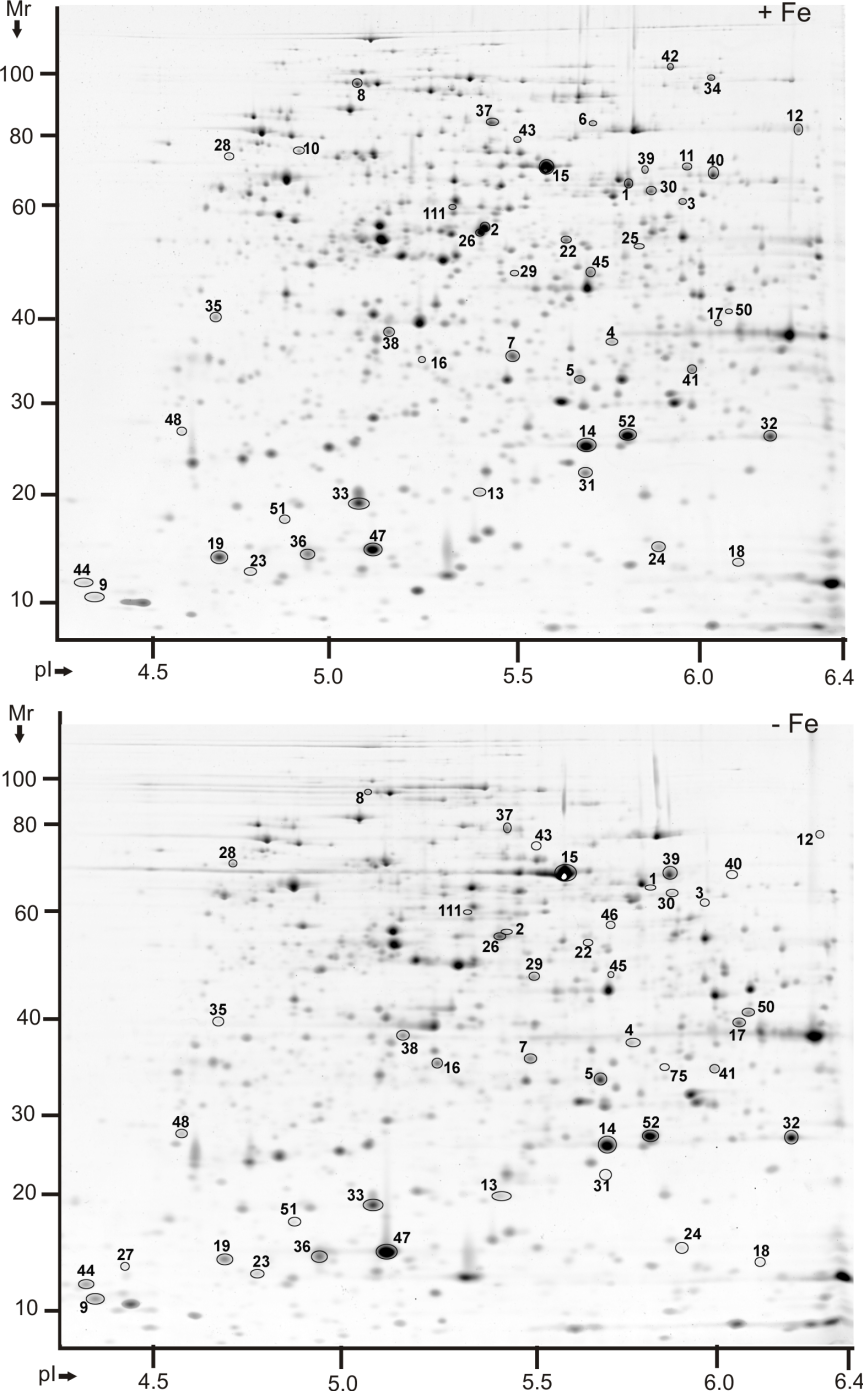
**Protein display in 2D gels of *Y. pestis *KIM6+ cytoplasmic fractions in the pI range 4-7 (-Fe *vs. *+Fe conditions)**. Proteins were derived from cell growth in the presence of 10 μM FeCl_3 _at 26°C (top) or the absence of FeCl_3 _at 26°C (bottom). Gels (20 × 25 cm) were stained with CBB, with four gel replicates representing each group, and subjected to differential display analysis using the software Proteomweaver v.4.0. Protein assignment to a spot required validation by MS data from at least two representative gels. The denoted spot numbers are equivalent to those listed in Table 3 with their '-Fe *vs. *+Fe' protein abundance ratios and other data.

a) spot number as denoted in Figure 4; b) protein accession number and locus tag as listed in *Y. pestis *KIM genome database (NCBI); c) gene name and protein description from the KIM database or a conserved *E. coli *K12 ortholog http://www.ecocyc.org, if >65 pct. sequence identity; d) subcellular localization based on PSORTb data: CY, cytoplasm; ML: multiple localizations; PP, periplasm; U: unknown; e) proven or putative regulation by Fur or a Fur-dependent small RNA (e.g. RyhB); f) highest Mascot score for a protein from LC-MS/MS or MALDI data; g) Vs (-Fe): average spot volume (n ≥ 3) in 2D gels for iron-depleted growth conditions at 26°C as shown in Figure 4; h) Vs (+Fe): average spot volume (n ≥ 3) in 2D gels for iron-supplemented growth conditions at 26°C; i) spot volume ratio (-Fe/+Fe) at 26°C, N.D.: not determined; -: no spot detected; j) two-tailed t-test p-value for spot abundance change at 26°C, 0.000 stands for < 0.001; k) average spot volume ratio (-Fe/+Fe) at 37°C; additional data for the statistical spot analysis at 37°C are part of Additional Table 1.

### *Y. pestis *iron acquisition systems

Proteomic profiling of characterized *Y. pestis *iron/siderophore and heme transporters (Ybt, Yfe, Yfu, Yiu and Hmu) was in good agreement with negative regulation of the respective operons by Fur and iron [15,16,20,49,50]. The subscript number following a protein name represents the spot number displayed in Figures 1, 2, 3 and 4, and is also denoted in the left-most column of Tables 1, 2 and 3. Periplasmic binding proteins of four of the ABC transporters (YfeA_#68_, YfuA_#65_, YiuA_#82 _and HmuT_#56_; Figures 1 and 2) were increased in abundance in iron-starved cells. The integral IM proteins YbtP and YbtQ were identified from streaky 2D spots of the usb-MBR fraction of iron-depleted cells, but could not be differentially quantitated. Two of these five transporters have an OM receptor responsible for iron/yersiniabactin or heme uptake (Psn_#102 _and HmuR_#95_, respectively; Figure 3), both of which were increased in iron-starved cells. Y0850_#96 _(Figure 3) is hypothesized to be a TonB-dependent OM receptor with Fe^3+^/siderophore uptake activity. This protein was also more abundant in iron-depleted cells. Detection in the usb-MBR fraction, its M_r _of *ca. *75-85 kDa and the presence of a highly conserved Fur-box upstream of the gene's transcriptional start site (AATGATAATTGATATCATT, -100 to -82) with a position weight matrix score of 13.2 using the patser-matrix tool [51] further supported the assignment as a Fur-regulated TonB-dependent OM receptor. Fur_#18 _was also detected in the cytoplasm, but not altered in abundance (Figure 4).

Abundance increases in iron-starved cells were observed for the multifunctional yersiniabactin synthase subunits HMWP1 and HMWP2 (products of the *irp1 *and *irp2 *genes, respectively) and other enzymes contributing to yersiniabactin biosynthesis (YbtS_#73_, YbtT_#75_, YbtE_#76 _and YbtU_#74_). The high M_r _proteins HMWP1 and HMWP2 were reliably quantitated only from SDS-PAGE gels (data not shown). The *ysu *locus encodes an OM receptor (YsuR/Y2633), an ABC transporter (Y2634-Y2637) and a suite of siderophore biosynthetic enzymes (Y2638-Y2641). Two subunits, Y2638_#78 _(Figure 1) and YsuR_#104 _(Figure 3) were of low abundance and detected only in iron-starved cells at 26°C. Biosynthesis of a Ysu siderophore has not been proven, and a siderophore biosynthetic pseudogene precedes the *y2633-y2637 *locus [18]. The OM β-barrel ferrichrome receptor FcuA_#103 _(Y2556) was identified as a protein of moderate abundance in usb-MBR fractions at 26°C (Figure 3) and 37°C, but not significantly altered in abundance comparing -Fe *vs. *+Fe conditions. Many membrane proteins ascribed to have putative functions in iron transport were not detected, *e.g. *the OM receptors Y3948 and IutA/Y3385 and the transport systems FitA-D (Y4043-Y4046), Y2837-Y2842 and FepB/Y3477. Our data support the notion of a hierarchy of iron (Fe^3+^)/siderophore transporters [15], with the Ybt and Yfe systems being dominant compared to the Yfu, Yiu and Hmu systems. Periplasmic subunits of two ferrous iron (Fe^2+^) transporters, EfeO/Y2451 and Y2368, were also profiled in 2D gels (Figure 1). The low M_r _protein Y2368_#72 _was increased in iron-starved cells at 37°C. The tripartite Fe^2+ ^transport protein EfeO_#77 _was increased in abundance in iron-starved cells at 26°C.

### The energy metabolism of *Y. pestis *is affected by iron starvation

Lower growth rates of *Y. pestis *in deferrated medium followed by growth arrest at OD_600_s between 0.5 and 0.9 suggest perturbations of energy generation pathways. Many oxidoreductive processes are catalyzed by enzymes containing Fe-S clusters or heme, and we sought to understand the consequences of limited iron availability as it pertains to the *Y. pestis *energy metabolism. The EcoCyc database http://www.ecocyc.org with its extensive data on *E. coli *energy metabolic pathways and iron cofactors of proteins was a useful resource in this context. *Y. pestis *aconitases A and B (AcnA_#34 _and AcnB_#8_; Figure 4) have functions in the TCA cycle and were decreased in abundance or detected only in iron-starved cells. So were subunits of two other TCA cycle enzymes harboring Fe-S clusters (SdhA_#43 _and FumA_#11_; Figure 4). Some TCA cycle enzymes devoid of Fe-S clusters were decreased at moderate levels under -Fe conditions (IcdA_#26_, SucA_#42_, SucD_#41 _and SucB_#111_; Figure 4). Strongly decreased abundances were denoted for AceA_#2 _and AceB_#1 _(Figure 4), enzymes which catalyze the glyoxalate bypass reaction of the TCA cycle and are regulated by the catabolite repressor protein (CRP). Glycerol kinase, also regulated by CRP, was more moderately decreased in iron-starved cells (GlpK_#3_, Figure 4). GlpK catalyzes the rate-limiting step of glycerol utilization and feeds its metabolites into the glycolytic pathway. CRP_#91 _itself was identified with low abundance in the periplasmic fraction (Figure 2). In summary, the data suggested reduced pyruvate metabolism via the citrate cycle when iron resources are exhausted in *Y. pestis *cells. Aconitase activity assays supported this assumption; the reaction rates were 2.8-fold and 2-fold higher in lysates derived from iron-replete cells *vs. *those from iron-starved cells at 26°C (stationary and exponential phase, respectively; Table 4).

**Table 4:** Reaction rates for four *Y. pestis *enzyme classes comparing -Fe *vs. *+Fe conditions

	Reaction rate^a)^ (nmol min-1 mL-1); (U mL-1)^b)^			Reaction rate^a)^ (nmol min-1 mL-1); (U mL-1)^b)^		
**Enzyme**	**+Fe, exp, n = 4^e)^**	**-Fe, early, n = 5^e)^**	**p-value^f)^**	**+Fe, stat, n = 4^e)^**	**-Fe, late, n = 5^e)^**	**p-value^f)^**

Aconitase ^c)^	2.31	1.14	0.019	4.98	1.82	0.008

Pyruvate oxidase ^c)^	167.5	1307	0.0001	463.0	2405	0.0001

Catalase ^d)^	82.5	31.8	0.0001	93.4	29.0	0.0001

Superoxide dismutase ^d)^	887.8	426.9	0.002	448.5	312.5	0.234

^a) ^Spectrophotometric assays in 96-well microtiter plates were used for the determination of enzyme reaction rates. Total protein concentrations in crude cell lysates were the same for all samples used in a given enzyme assay: aconitase, 0.5 mg/mL; pyruvate oxidase, 0.4 mg/mL; catalase, 0.15 mg/mL; superoxide dismutase, 1.1 μg/mL. ^b) ^Units ml^-1 ^was the definition for the superoxide dismutase reaction rate. All assays were performed in duplicate. ^c) ^Reaction rates from the linear part of the slope of the absorbance change over time. ^d) ^Reaction rates from endpoint assays. ^e) ^Number of biological replicates of cell lysates (n); exp: abbreviation for exponential, early: early growth phase equivalent to exp. phase (-Fe); average OD_600 _= 0.66 (+Fe) and OD_600 _= 0.47 (-Fe); stat: abbreviation for stationary growth phase, late: late growth phase equivalent to stat. phase (-Fe); average OD_600 _= 2.0 (+Fe) and OD_600 _= 0.81 (-Fe). True exponential and stationary growth phases were not observed for cell cultures in iron-free media. ^f) ^p-values were calculated from to comparison of reaction rates (+ Fe *vs*. -Fe) using a two-tailed t-test method.

The question arose whether iron-starved *Y. pestis *cells activated a different metabolic route of pyruvate degradation able to produce reducing equivalents (NADH and UQH_2_) for the electron transport chain. Pyruvate oxidase (PoxB) degrades pyruvate to acetate and is a flavin-dependent, iron-independent enzyme that generates UQH_2 _[52]. The pyruvate oxidase pathway indeed appeared to be important, as judged by the strong abundance increase of PoxB_#39 _(Figure 4) under -Fe conditions. The flavin cofactor may be recruited from redox activities of two flavodoxins. FldA3_#44 _was quite abundant and moderately increased in iron-depleted cells (Figure 4). FldA was identified in faint 2D spots and not reproducibly quantitated. PoxB activity measurements revealed excellent correlation between enhanced abundances and increased reaction rates in iron-starved cells. PoxB activities were 5.3-fold and 7.8-fold higher in lysates of iron-starved cells than in lysates of iron-replete cells at 26°C (stationary and exponential phase, respectively; Table 4).

Electron transport chains are localized in the IM, a fact that compromised the analysis of subunits of these IM protein complexes in 2D gels. NuoCD_#99_, a peripheral membrane protein of the NADH:ubiquinone oxidoreductase, was moderately decreased in abundance in iron-depleted cells (Figure 3). The *E. coli *NuoCD sub-complex is important for binding of some of the six Nuo-integrated Fe-S clusters [53]. Subunits of Fe-S cluster proteins with roles in two anaerobic energy metabolism branches were also less abundant in iron-depleted cells. This pertained to PflB_#37 _and YfiD_#19_, proteins of the formate-pyruvate lyase complex, and FrdA_#6_, which is part of the terminal electron acceptor fumarate reductase (Figure 4). Decreased abundances of metabolically active Fe-S cluster enzymes were a notable feature of iron-starved *Y. pestis *proteome profiles, while the abundance and activity of PoxB suggested that this enzyme was important to maintain the aerobic energy metabolism and iron cofactor-independent generation of UQH_2 _in iron-deficient *Y. pestis *cells.

### Oxidative stress response in *Y. pestis *under iron starvation conditions

Oxidative stress is caused by various oxygen radicals and H_2_O_2_, and catalyzed by redox enzymes in non-specific reactions. While the presence of free intracellular iron aggravates oxidative stress via the Fenton reaction, it is mitigated by cytoplasmic proteins that scavenge free iron, *e.g. *Dps and the ferritins FtnA and Bfr [54]. The question arose how aerobically growing, iron-deficient *Y. pestis *cells coped with oxidative stress. One of the main *E. coli *global regulators of the oxidative stress response, the Fe-S cluster protein SoxR, is not encoded in the *Y. pestis *genome [2]. The other global oxidative stress response regulator is OxyR. OxyR_#4 _(Figure 4) was not altered in abundance in *Y. pestis *comparing -Fe and+Fe conditions. Among the enzymes deactivating H_2_O_2 _and oxygen radicals are catalases/peroxidases and superoxide dismutases (SODs). *Y. pestis *produces two catalases with heme cofactors in high abundance. KatE_#40 _(Y2981) was predominantly expressed at 26°C (Figure 4) and KatY_#12 _(Y0870) at 37°C. Cytoplasmic SODs include SodB_#31_, which has an iron cofactor, and SodA_#52_, which has a manganese cofactor (Figure 4). Periplasmic SodC_#84 _has a copper/zinc cofactor (Figure 2). Iron availability-dependent patterns of abundance changes reminiscent of enzymes with functions in energy metabolism were observed. Only the iron-dependent proteins KatE, KatY and SodB were strongly diminished in abundance in iron-depleted cells (Table 3). We also determined overall catalase and SOD activities. Catalase reaction rates were 3.2-fold and 2.6-fold higher in lysates derived from iron-replete *vs. *iron-starved cells at 26°C (stationary and exponential phase, respectively; Table 4). SOD reaction rates were 2-fold higher in the exponential phase, but not significantly altered in the stationary phase (Table 4). This data was in good agreement with differential abundance data, although individual activities of SodA, SodB and SodC could not be discerned with the assay.

AhpC_#14_, Tpx_#33_, TrxB_#38 _and Gst_#32_, all of which are involved in redox homeostasis and deactivation of oxidative compounds, were similarly abundant in iron-rich *vs. *iron-starved *Y. pestis *cells (Figure 4). These enzymes contain either disulfide- or flavin-based redox centers. Dps_#24_, an iron-scavenging protein important for the protection and repair of DNA under general stress conditions, was moderately decreased in abundance under -Fe conditions, but only at 26°C. The OxyR H_2_O_2_-response system of *E. coli *was reported to restore Fur in its ability to repress gene expression in the presence of iron by increasing the protein's synthesis during oxidative stress [32], a mechanism that may be applicable to *Y. pestis*. We conclude that the bacterium adjusts its repertoire of oxidative stress response proteins when iron is in short supply, by reducing the abundance of those proteins that require iron cofactors for functional activity.

### Iron storage and iron-sulfur cluster biosynthesis in *Y. pestis*

High concentrations of free Fe^3+ ^are toxic to bacterial cells and require sequestration by proteins. FtnA and Bfr are the main cytoplasmic iron storage proteins. FtnA_#36 _was slightly increased in iron-depleted cells at 26°C (Figure 4), but not at 37°C. Bfr_#51 _(Figure 4) was of considerably lower abundance than FtnA and not significantly changed in abundance comparing -Fe *vs. *+Fe conditions. The *Y. pestis *KIM genome harbors two gene clusters orthologous to those of the *E. coli isc *and *suf *operons (*y1333-y1341 *and *y1934-1939*, respectively). The gene products are responsible for Fe-S cluster assembly under normal growth and stress conditions, respectively. *E. coli sufABCDSE *expression was reported to be controlled by the regulators OxyR (oxidative stress) and Fur (iron starvation) [55]. Protein profiling revealed that the *Y. pestis *Suf proteins were considerably increased or detected only in iron-depleted cells (SufC_#69 _and SufD_#70_, Figure 1; SufA_#27_, SufB_#28 _and the cysteine desulfurase SufS_#29_; Figure 4). Four *Y. pestis *Isc subunits (IscS, NifU, HscA and HscB) were detected at very low abundance in cytoplasmic fractions. The cysteine desulfurase IscS_#20 _and the chaperone HscA_#21 _were diminished in abundance in iron-starved cells at 37°C (Table 3). In contrast, an ortholog of the *E. coli *essential respiratory protein A (ErpA_#9_) was increased in abundance in iron-starved cells, particularly at 26°C (Figure 4). This low M_r _Fe-S cluster protein was proposed to serve in the transfer of Fe-S moieties to an enzyme involved in isoprenoid biosynthesis [56]. Its expression was described to be under the control of *E. coli *IscR, the regulator of the *isc *gene locus. However, the abundance changes of *Y. pestis *ErpA (-Fe *vs*. +Fe) resemble those of the Suf rather than the Isc subunits.

The question arose whether sulfur-mobilizing proteins were also altered in abundance comparing -Fe and +Fe conditions, in order to support a Fe-S cluster rebalancing effort among proteins localized in the *Y. pestis *cytoplasm. Periplasmic binding proteins of sulfate/thiosulfate ABC transporters (Sbp, CysP) were not altered in abundance (data not shown). The flavoprotein subunit of sulfate reductase (CysJ_#10_) was strongly decreased in abundance under iron-limiting conditions (Figure 4). CysI, the Fe-S cluster subunit, was not detected. Taurine dioxygenase however (TauD_#50_, Figure 4), which utilizes aliphatic sulfonates as a sulfur source, was increased in iron-starved *Y. pestis *cells. The *E. coli *dioxygenase TauD seems to require iron for activity according to a note in the EcoCyc database. Whether the activity of TauB is linked to Fe-S cluster biosynthesis or repair remains to be shown. In summary, our data supported a functional role of the *Y. pestis *Suf system in Fe-S cluster assembly when cells are deprived of iron. Data related to CysIJ suggested that Fe-S cluster proteins active in pathways unrelated to energy metabolism were also down-regulated upon intracellular iron starvation.

### Protein abundance changes less obviously linked to iron homeostasis

**Figure 5:**
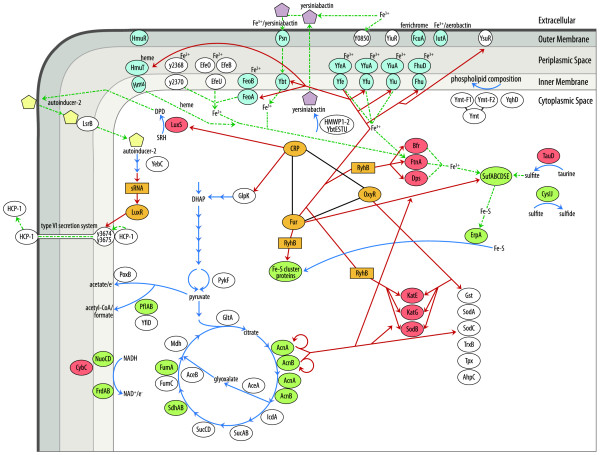
**Iron homeostasis in *Y. pestis***. The center of the schematic depicts a network of regulators (orange color), known or potentially involved in iron homeostasis. Details are provided in the text. CRP (carbon metabolism); OxyR (oxidative stress); Fur and small RNAs like RyhB (iron homeostasis). Red lines/arrows show which genes (or mRNAs) are controlled by these regulators. Additional arrows symbolize enzymatic reactions (blue line) or small molecule transport processes (dotted green line). The lower/left side of the schematic depicts components of the energy metabolism. It includes glycolytic steps from dihydroxyacetone phosphate (DHAP) to pyruvate, the TCA/glyoxalate bypass cycle and on the left side alternative pyruvate metabolism branches generating acetate or acetyl-CoA. Subunits of electron transport systems (NuoCD, FrdAB and CybC) are also displayed. The top/left side of the schematic pertains to quorum sensing. LuxS converts S-ribosylhomocysteine (SRH) to 4,5-dihydroxy-2,3-pentanedione (DPD) which is a precursor of autoinducer-2 (yellow pentagon). In *E. coli*, the autoinducer-2 is exported and imported via periplasmic LsrB into different cells followed by activation of LuxR via small RNA regulators. The precise functional role of YebC in quorum sensing is not known. LuxR influences the expression of virulence factors in pathogenic *E. coli *strains. The role of LuxR in the regulation of the type VI secretion system is speculative, but both iron starvation [73] and the T6SS [74] have been linked to quorum sensing in other organisms. In the upper part of the schematic, iron transporter subunits are placed according their predicted or known subcellular localizations. Transporters with a blue color background are known to be functional in *Y. pestis*. On the center/right side, iron storage proteins, the Suf Fe-S cluster assembly system and putative sulfur-mobilizing enzymes (TauD and CysIJ) are displayed. The bottom/right part of the schematic features oxidative stress response proteins. Finally, the top/right part of the schematic displays the flea survival factor Ymt and its fragments, as well as the protein YqhD. These proteins may be implicated in the enzymatic modifications of IM phospholipids. Proteins with a red and green background harbor iron/heme and Fe-S cluster cofactors, respectively.

Iron is an essential cofactor for many cellular processes, and a network of global regulators (CRP, OxyR and Fur/RyhB; Figure 5) are affected by or implicated in responses to iron deficiency. We expected to detect protein abundance changes less obviously linked to iron homeostasis. S-ribosylhomocysteinase (LuxS_#13_) is an enzyme of central importance in the activated methyl cycle and plays a role in autoinducer 2-mediated quorum sensing in *E. coli *[57]. The enzyme harbors tetrahedrally coordinated Fe^2+ ^in its catalytic center. LuxS was moderately increased in iron-depleted cells at 26°C (Figure 4). In contrast, LsrB_#87 _whose *E. coli *ortholog facilitates periplasmic transport of the autoinducer 2 following cellular re-uptake was decreased in abundance in iron-starved cells (Figure 1), similar to YebC_#35_, a protein hypothesized to be involved in quorum sensing regulation [58]. *Y. pestis *has been shown to produce the autoinducer 2, although genes controlled by this system have not been identified [59]. Slightly increased abundances of four subunits of a putative type VI secretion system (T6SS) were also observed in iron-deficient *vs. *iron-rich cells. The proteins HCP1_#47 _and Y3675_#48 _(Figure 4), Y3676_#86 _(Figure 1) and Y3674_#110 _(Figure 3) were not at all detected in *Y. pestis *protein profiles at 37°C. The T6SS is temperature-regulated. The flea survival factor Ymt_#15 _(Figure 4) was moderately increased in iron-starved cells at 26°C. It was one of the most abundant proteins in cells grown at 26°C. N- and C-terminal fragments of Ymt, each *ca. *30 kDa in size and with a single cleavage site between V_300 _and I_304 _(Ymt_#16 _and Ymt_#17_, respectively; Figure 4), were also increased under -Fe *vs. *+Fe conditions. There is no evidence for a connection between the functional roles of Ymt or the T6SS and the iron starvation response.

Periplasmic subunits of ABC transporters for amino acids, sugars and phosphate, various diffusion porins of the OM allowing passage of nutrients into the periplasm, and various amino acid tRNA-synthetases and enzymes implicated in amino acid biosynthesis were significantly increased in abundance in iron-replete cells. These observations were consistent with the notion that *Y. pestis *cells grown to stationary phase under +Fe conditions were depleted of various nutrients and induced the expression of high affinity transport systems for their import into the cell. Examples were the phosphate-specific OM porin PhoE_#109 _(Figure 3) and the periplasmic maltose-binding protein MalE_#53 _(Figure 1), each of which was much more abundant under +Fe *vs. *-Fe conditions.

## Discussion

### Regulation of iron homeostasis

Fur in *Y. pestis*, as in many other Gram-negative bacteria, is a central transcriptional regulator responding to the cellular iron status [20,50], as indicated in the schematic of Figure 5. Many iron uptake systems are transcriptionally repressed during iron-replete growth conditions to reduce accumulation of intracellular iron. Evidence has emerged that small RNA regulators are implicated in bacterial stress responses [22]. These small RNAs act by base-pairing with specific mRNAs whose translation they stimulate or inhibit in the presence of a unique protein, the RNA chaperone Hfq. A small RNA of 90 nucleotides determined to regulate genes involved in iron homeostasis in *E. coli *[23] and *Pseudomonas aeruginosa *[24] was termed RyhB. It is negatively regulated by Fur and was shown to down-regulate the translation of many of the same iron-dependent enzymes we detected as decreased in iron-starved *Y. pestis *cells (SdhA, AcnA, FumA, FrdA, SodB, KatE and KatY) [23]. We hypothesize that one or both of the conserved *Y. pestis *homologs of RyhB [22] co-regulate *Y. pestis *iron homeostasis and selectively decrease translation of mRNAs whose protein products depend on or store iron, as illustrated in Figure 5. Such a mechanism may restrict the use of scarce intracellular iron to processes pivotal to bacterial survival. Some of the encoding genes (*e.g. ftnA*, *katE *and *sodB*) may also be positively controlled by Fur as suggested by Yang *et al. *[35]. Gel shift assays revealed binding of recombinant Fur to promoter regions upstream of the genes *ftnA *and *katE *[20].

Several of the enzymes decreased in abundance in iron-deficient *Y. pestis *harbor Fe-S clusters. Expression of the respective genes did not appear to be altered under conditions sequestering or depleting iron in *Y. pestis *according to two DNA microarray studies [33,35] and suggests post-transcriptional mechanisms. The involvement of RyhB in controlling the abundances of proteins with iron cofactors when cells are iron-deficient needs to be verified. Since our data were derived from proteomic comparisons of *Y. pestis *cells harvested at different cell densities (OD_600_s of ~2.0 for stationary phase cells *vs. *OD_600_s of ~0.8 for growth arrested, iron-starved cells), the argument can be made that population density differences account for some of the protein abundance changes. Unpublished data (Pieper, R.) and a previous study analyzing the *Y. pestis *periplasmic proteome in the context of two growth phases [39] allow us to largely refute this notion. Among the proteins with iron or Fe-S cofactors, only PflB and KatE were increased in stationary *vs. *exponential phase proteomic profiles with ratios comparable to those observed in iron-rich *vs. *iron-starved cells. FtnA and Bfr are iron storage proteins and, via regulation by RyhB, were reported to be quantitatively decreased when iron supplies are limited in *E. coli *[23]. Our data on the FtnA and Bfr orthologs of *Y. pestis *were not consistent with the results of the aforementioned studies, nor with two *Y. pestis *transcriptional profiling studies where increased *bfr *expression and, in one case, decreased *ftnA *expression were reported for iron-limiting growth environments [33,35].

Post-transcriptional regulatory functions in iron-deficient cells have also been attributed to aconitases. In fact, eukaryotic AcnA has been termed iron-responsive protein 1 (IRP-1) [60]. Apo-enzyme versions of *E. coli *aconitases stabilize their cognate mRNAs and influence the expression of *sodA*. AcnA enhanced *sodA *transcript stability and was induced by iron starvation and oxidative stress in *E. coli *[61,62]. These findings could not be easily reconciled with our data onAcnA and AcnB abundance changes in *Y. pestis*. AcnA and AcnB were decreased in abundance, as were the combined aconitase activities, in iron-depleted cells. SodA abundance was not significantly affected by either growth phase [39] or iron depletion. The response of *Y. pestis *to iron starvation and cellular stress resulting from the loss of this metal ion seems to implicate a network of regulators, as presented in Figure 5. Indeed, functional relationships between Fur and OxyR [32], Fur and CRP [31] and Fur and apo-aconitases [62] were previously reported for *E. coli*.

### Iron starvation stress responses

Numerous *E. coli *genes encoding oxidative stress response proteins are co-regulated by SoxR, Fur and OxyR according to information in the EcoCyc database. The OxyR H_2_O_2_-response system restored Fur repression in iron-replete media during oxidative stress in *E. coli *[32], a mechanism that we think is also relevant in *Y. pestis*. Strong abundance decreases in iron-starved *Y. pestis *cells were observed for three iron-dependent proteins, SodB, KatE and KatY. The three enzymes detoxify peroxides and radicals formed during oxidative stress. Proteins with similar functions but cofactors other than iron (*e.g. *SodA and AhpC) were not markedly changed in abundance. Functional assays supported such proteomic data; SOD activities in iron-depleted cells dropped markedly less than catalase activities. In conclusion, our data strongly support the notion that *Y. pestis *adapts its repertoire of oxidative stress response enzymes by limiting the expression of iron cofactor-dependent enzymes, when iron is in short supply. The coordination of bacterial responses to iron limitation and the defence against oxidative stress was proposed earlier [63].

### Iron acquisition systems

All *Y. pestis *biovars have several proven iron acquisition systems, and transcriptional control by Fur has been demonstrated [18,64]. The genes and operons for putative iron transporters (*e.g. *Ysu, Fit, Fhu, Iuc, Has) also feature conserved 19-nt Fur-binding sites to which recombinant Fur binds [20]. Our data indicated that none of the abovementioned iron transporters were expressed at levels similar to those observed for subunits of proven iron transport systems (Ybt, Yfe, Yfu, Yiu and Hmu) under iron-deficient conditions. Two microarray studies, however, reported increased transcript abundances for many of the putative iron transporters when iron was complexed with dipyridyl [35] or sequestered by iron-binding proteins in blood plasma [33]. 2D gel analysis has known limitations pertaining to protein detection sensitivity and the resolution of hydrophobic IM-localized proteins, *e.g. *many nutrient transporters. Except Ysu subunits, unproven iron transporters were also not profiled employing a peptide-based LC-MS/MS analysis approach with *Y. pestis *lysates [47,65]. These lysates were derived from iron-replete growth conditions. Only functional iron transporters are presented in the schematic of Figure 5 and appear to follow a hierarchy of importance in the order of Ybt, Yfe (each important for virulence in a bubonic plague model), Yfu and Yiu [15]. The delivery of Fe^3+ ^or Fe^2+ ^from the extracellular milieu to periplasmic binding proteins of the ABC transporters Yfe, Yfu and Yiu is unclear, although a YiuR surface receptor was expressed according to our data. The Hmu transporter acquires heme from blood plasma proteins such as myoglobin, hemoglobin and hemopexin [16]. Three Fe^2+ ^transport systems (EfeUOB, Y2368-Y2370 and FeoAB, Figure 5) were shown to be functional in either *Y. pestis *[17] or other bacteria [66-68]. We identified the subunits EfeO and Y2368 as periplasmic proteins, and their abundance increases in iron-deficient cells appeared to be moderately temperature-dependent. There is no evidence to date for their regulation by Fur. FeoB was recently identified in *Y. pestis *membrane proteome surveys [47,65]. A protein highly abundant in membrane fractions of iron-depleted *Y. pestis *cells but not characterized in the context of iron transport was the orphan TonB-dependent OM receptor Y0850. The protein is a candidate for Fur regulation and the contribution to iron uptake, but its exact function remains to be elucidated. A conserved Fur box upstream of the gene and sequence similarity of Y0850 to *Bordetella bronchiseptica *BfrA and *Campylobacter coli *CfrA [69,70] were established. Our proteomic surveys did not support the activation of specific iron uptake pathways at only one of the physiologically relevant temperatures. Based on multivariate transcriptional profiling data for *Y. pestis *(28°C *vs. *37°C, iron-supplemented cell growth *vs. *iron sequestration in plasma), Carniel *et al. *[33] suggested that the Ybt system and the TonB protein are of particular importance for iron acquisition at 37°C.

### Fe-S cluster biosynthesis and energy metabolism in iron-starved *Y. pestis*

Growth of iron-depleted *Y. pestis *cells was arrested at an OD_600 _of ~0.8, indicative of the inability of iron-dependent enzymes to perform essential metabolic functions. In addition to the already discussed impact of iron depletion on oxidative stress response enzymes and aconitases, we explored how Fe-S cluster assembly systems and other energy metabolism enzymes were affected. Iron-sulfur clusters are critical to the function of many redox enzymes in bacteria [27]. Incorporation of Fe-S into proteins requires Fe-S cluster assembly systems, which were named Suf and Isc in *E. coli*. Our data showed that SufA, SufB, SufC and SufS, four of the six subunits of the Suf complex, were more abundant under iron starvation conditions. Regulation of the *Y. pestis suf *operon by Fur and a functional Fur-binding site were reported previously [20]. The cysteine desulfurase subunits of the Suf and Isc systems (SufS and CsdA, respectively) were quantitatively changed in opposite directions (-Fe *vs. *+Fe), suggesting that Suf functionally replaces Isc at the onset of iron starvation in *Y. pestis*. Mobilization of sulfur from cysteine appears to be catalyzed by SufS in *E. coli *[71]. The increased abundance of TauD, an enzyme that mobilizes sulfite from taurine, in iron-depleted *Y. pestis *cells was intriguing. TauD is a dioxygenase, harbors a Fe^2+ ^cofactor and was reported to be induced under sulfate starvation conditions in *E. coli *[72]. We speculate that TauD plays an accessory role in sulphur mobilization for Fe-S cluster assembly via the Suf pathway. Furthermore, the *Y. pestis *ortholog of a recently discovered Fe-S cluster protein ErpA was also increased under iron-limiting conditions. Since ErpA was proposed to transfer Fe-S clusters to apo-enzymes [56], we hypothesize that *Y. pestis *ErpA may perform such activities cooperatively with the Suf system. Transcriptional data on *erpA *and *tauD *expression changes for -Fe *vs. *+Fe growth conditions are not available. Mammalian hosts starve *Y. pestis *of iron and, therefore, the Suf complex constitutes a good target for inhibitory drug design.

Enzymes with Fe-S clusters in their catalytic cores, many of them in the TCA cycle, are also displayed in Figure 5. Although in different ratios, subunits of such enzyme complexes (*e.g. *FumA, SdhA, FrdA and CysJ) were invariably decreased in abundance in iron-starved *Y. pestis *cells. Most of these quantitative decreases appear to be unrelated to population density differences, because they were not observed in cells cultured to stationary *vs. *exponential phase in iron-replete PMH2 medium(Pieper, R., unpublished data). A decreased pyruvate metabolism rate should be the consequence of the loss of Fe-S cluster enzyme activities in the TCA cycle and may be followed by reduced production of ATP and NADPH reducing equivalents in the electron transport chain. Furthermore, a decreased turnover of citrate may lead to its accumulation in the cytoplasm, which could chelate iron and exacerbate iron starvation [30]. A highly interesting observation was the dramatic abundance and activity increase of PoxB in iron-starved *Y. pestis *cells, both at 26°C and 37°C. PoxB activity increases were independent of *Y. pestis *cell densities during growth in chemically defined media. *poxB *expression was reported to be moderately enhanced in *Y. pestis *cells grown in human plasma *vs*. LB media [33]. We suggest that the metabolism of pyruvate via the PoxB route compensates for reduced activities of Fe-S cluster enzymes in the TCA cycle. The pathway catalyzed by PoxB is iron-independent. The *E. coli *ortholog, a thiamin/flavin-dependent enzyme activated by binding to IM phospholipids, was shown to feed electrons directly from the cytosol to the respiratory chain [52]. To our knowledge, this is the first report linking enhanced PoxB activities in bacteria specifically to iron starvation. PoxB is a potential drug target in the context of intracellular pathogens surviving in environments where iron is sequestered.

## Conclusions

Proteomic surveys of *Y. pestis *subcellular fractions grown under iron-replete *vs. *iron-starved conditions supported the physiological importance of the iron acquisition systems Ybt, Yfe, Yfu, Yiu and Hmu. An uncharacterized TonB-dependent OM receptor, Y0850, was also highly abundant in iron-depleted cells, appeared to be Fur-regulated and may participate in iron uptake. Numerous enzymes harboring iron and Fe-S cluster cofactors were significantly decreased in abundance in iron-starved cells, suggesting a regulatory process shifting the metabolism of *Y. pestis *to iron-independent pathways when the supply of this metal ion is limited. Small Fur-regulated RNAs termed RyhB in *E. coli *may be involved in this process. Finally, this study revealed biochemical pathways likely essential for the iron starvation response in *Y. pestis*. Examples are the energy metabolism via the pyruvate oxidase route and Fe-S cluster assembly mediated by the Suf system.

## Abbreviations

2D: 2-dimensional gel electrophoresis; CBB: Coomassie Brilliant Blue G250; Fe-S: iron-sulfur; IM: inner membrane; usb-MBR: urea/thiourea/amidosulfobetaine-14-extracted membrane; IPG: immobilized pH gradient; OM: outer membrane; SOD: superoxide dismutase; T3SS: type III secretion system; T6SS: type VI secretion system; TMD: transmembrane domain; V_S_: spot volume.

## Competing interests

The authors declare that they have no competing interests.

## Authors' contributions

RP: primary role in designing the study, analyzing and interpreting the data, performing the enzyme assays, writing the article; STH: quantitative and bioinformatic data analysis, database queries, generation of Figures and Tables for the article; PPP: sample preparation, 2D gel experiments and proof-reading; DJC: acquisition of the LC-MS/MS data; HA: acquisition of the MALDI-MS data; RDF: generated the framework for the performance of this study; RDP: major role in the design and initial experiments of the study, biological interpretation of the data, writing parts of the article and its review; SNP: major role in the biological data interpretation and the review of the article.

## Supplementary Material

Additional file 1 ***Yersinia pestis *growth curves in PMH2 medium**. Growth curves (OD_600_) are displayed in graphical form for *Y. pestis *KIM6+ cell cultures in iron rich and iron-depleted media, at 26°C and at 37°C. Click here for file

Additional file 2 **Comprehensive list of differentially displayed *Yersinia pestis *proteins comparing iron-replete and iron starvation conditions**. A variety of qualitative and quantitative data are provided for differentially displayed proteins derived from + Fe *vs*. -Fe growth conditions, from cell cultures at 26°C and at 37°C. Click here for file

Additional file 3 **Comprehensive list of MS and MS^2 ^data for *Y. pestis *KIM6+ proteins**. For all proteins listed in the Tables 1, 2 and 3 and in the Additional File 2, MS and MS^2 ^data were parsed from MALDI-TOFTOF and LC-nESI-LC-MS/MS datasets. Click here for file
