# Supplementary material for: Proteomic analysis of iron acquisition, metabolic and regulatory responses of Yersinia pestis to iron starvation
Source: BMC Microbiol. 2010 Jan 29;10:30. doi: 10.1186/1471-2180-10-30 (PMC2835676; doi:10.1186/1471-2180-10-30)
Supplement: Additional file 1 — Yersinia pestis growth curves in PMH2 medium. Growth curves (OD600) are displayed in graphical form for Y. pestis KIM6+ cell cultures in iron rich and iron-depleted media, at 26°C and at 37°C. [file 1471-2180-10-30-S1.DOC]

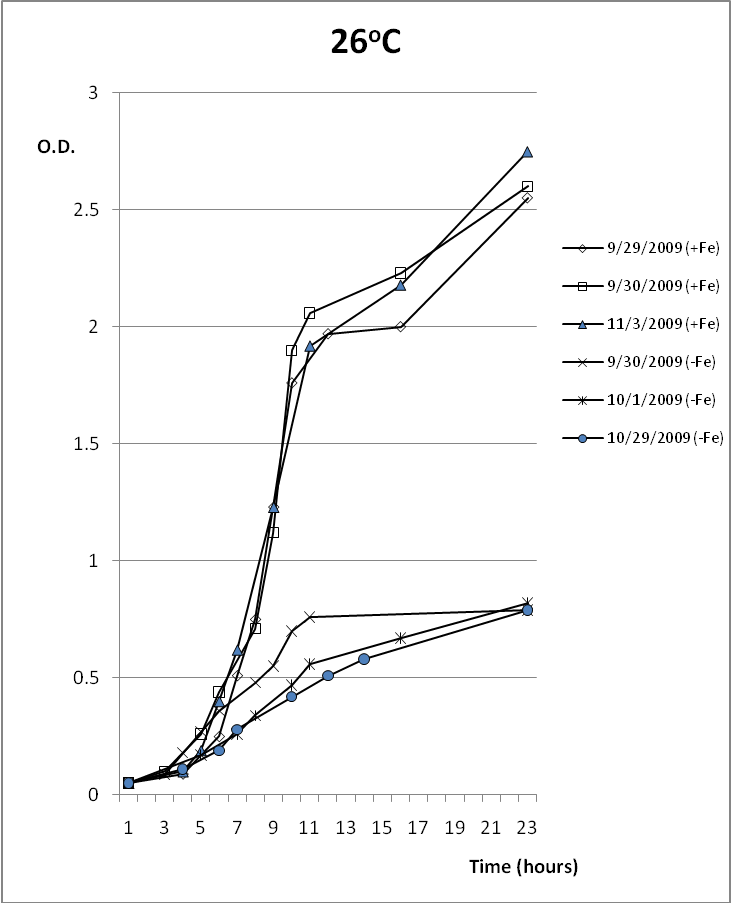


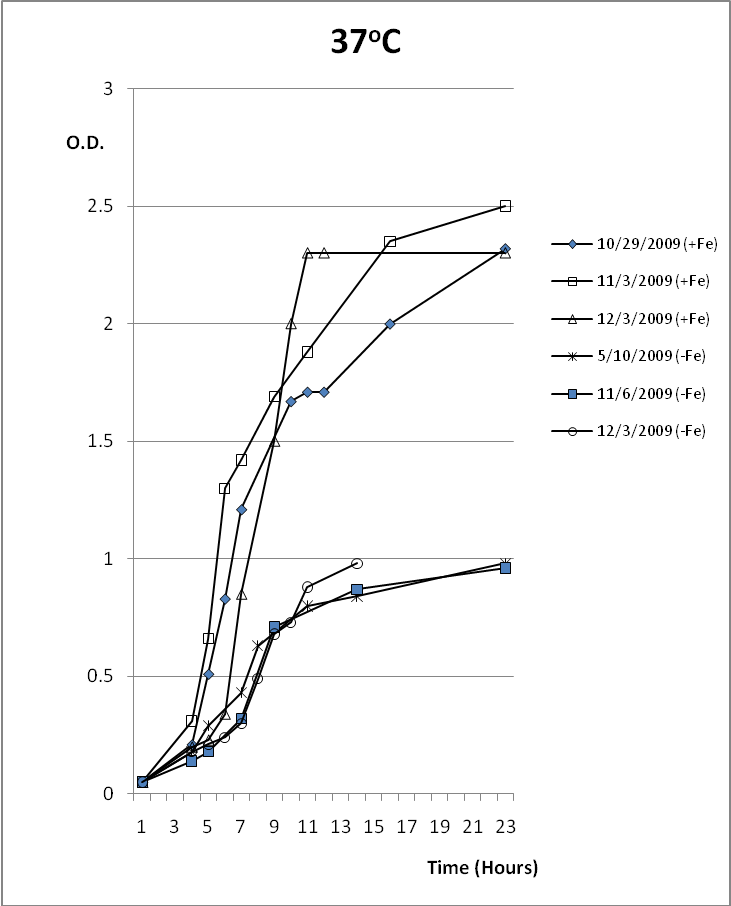


**Supplemental information.** *Yersinia pestis* KIM6+ cells were grown at 26°C and 37°C in chemically defined media (PMH2) in shaker flasks (225 rpm), either in the presence of 10 μg/mL FeCl_3_ or, after removal of residual iron from PMH2 using Chelex 100 resin, in the absence of FeCl_3_. Cultures were started at an OD_600_ of 0.05. The legends indicate which growth curves pertain to suspension cultures with and without 10 μg/mL FeCl_3_ (+Fe and –Fe, respectively). The –Fe cultures did not display exponential and stationary phases which are typical for growth curves in complete PMH2 media.
